# 

**Published:** 1792

**Authors:** 


### CATALOGUE OF BOOKS.

1. SURGICAL TRACTS, by the late *J. O. Juſtamond*, F. R. S. Surgeon to the Weſtminſter Hoſpital: conſiſting of, 1. Outlines of the Hiſtory of Surgery, from the earlieſt Antiquity of the Art, pointing out the particular Improvements, and fixing them where due. 2. An Eſſay on Inflammation and Abſceſs, with their proper Modes of Treatment in different Parts of the Body. 3. A Diſſertation on the Effects of Motion and Reſt, and their

Application to the Purpofes of Surgery; from the French Prize Memoir, by M. *David*, with copious additional Annotations on the original Text. 4. Obfervations on Counter Strokes, and an Account of their various Confequences, Treatment, &c. from the fame. 5. On the Methods employed in treating Cancerous Difeafes, including Remarks on the Cure of Indurations of the Breaft. The whole collected, and interfperfed with occafional Notes and Obfervations, by *William Houlfton*, Member of the Corporation of Surgeons, Fellow of the Society of Antiquaries, and of the Medical Society of London. 4to. *Cadell*, London, 1790.

2. Experiments and Obfervations on the Horley-Green Spa, near Halifax. To which is added a fhort Account of two other Mineral Waters in Yorkfhire. By *Thomas Garnett*, M. D. late Prefident of the Royal Phyfical and Natural Hiftory Societies, and Member of the Royal Society at Edinburgh. 8vo. *Knott*, London, 1790.

3. The Anatomical Inftructor; or an Illuftration of the modern and moft approved Methods of preparing and preferving the different Parts of the human Body, and of Quadrupeds, by Injection, Corrofion, Maceration, Diftention,

Articu-

Articulation, Modelling, &c. with a Variety of Copper Plates. By *Thomas Pole*, Member of the Corporation of Surgeons in London. 8vo. *Darton*, London, 1790.

4. A Treatife on the Difeafe commonly called Angina Pectoris. By *William Butter*, M. D. Fellow of the Royal College of Phyficians, and Member of the Medical Society, both of Edinburgh. 8vo. *Johnfon*, London, 1791.

5. Tranfactions of the Linnean Society. Vol. I. 4to. *White*, London, 1791.

6. Select Evidences of a fuccefsful Method of treating Fever and Dyfentery in Bengal. By *John Peter Wade*, M. D. of the Hon East-India Company's Bengal Eftablifhment. 8vo. *Murray*, London, 1791.

7. A Treatife on the Cataract; with Cafes to prove the Neceffity of dividing the tranfparent Cornea, and the Capfule of the cryftalline Humour, differently, in the different Species of this Difeafe. By M. de *Wenzel*, Junior, Baron of the Holy Roman Empire, Phyfician of the Faculty of Nancy, and Regent Doctor of the Faculty of Medicine in the Univerfity of Paris. Tranflated from the French, with many additional Remarks. By *James Ware*, Surgeon. 8vo. *Dilly*, London, 1791.

8. A Plan

8. A Plan of a Charitable Inſtitution, intended to be eſtabliſhed upon the Sea Coaſt, for the Accommodation of Perſons afflicted with ſuch Diſeaſes as are uſually relieved by Sea-Bathing. By *John Latham*, M. D. Fellow of the Royal College of Phyſicians, and Phyſician to the Middleſex and Magdalen Hoſpitals, 8vo. *Longman*, London, 1791.

9. The great Importance and proper Method of cultivating and curing Rhubarb in Britain, for medicinal Uſes; with an Appendix. By Sir *William Fordyce*, M. D. F. R. S. 8vo. *Cadell*, London, 1792.

10. Pharmacopœia Collegii Regalis Medicorum Londinenſis. 8vo. Londini, 1791.

11 Pharmacopœia in uſum Noſocomii Mancunienſis. 12mo. Mancunii, 1791.

12. Diſſertatio Inauguralis de Rheumatiſmo acuto. Auctore *Carolo Angier*, Anglo. 8vo. Edinburgi, 1791.

13. Diſſertatio Inauguralis de Variolis. Auctore *Gulielmo Barrow*, Anglo. 8vo. Edin. 1791.

14. Diſſertatio Inauguralis de Epiſpaſticorum Uſu. Auctore *Thoma Bradley*, Anglo. 8vo. Edin. 1791.

15. Diſſertatio Inauguralis de Ictero. Auctore *Gulielmo Briggs*, Anglo. 8vo. Edin. 1791.

10. Diſ-

16. Dissertatio Inauguralis de Asthmate Spasmodico. Auctore *Joanne Clendining*, Hiberno. 8vo. Edin. 1791.

17. Dissertatio Inauguralis de Epilepsia. Auctore *Adamo Douglass*, Hiberno. 8vo. Edin. 1791.

18. Dissertatio Inauguralis de Dyspepsia. Auctore *Philippo Elliot*, Cambro-Britanno. 8vo. Edin. 1791.

19. Dissertatio Inauguralis de Somniis. Auctore *Josepho Gahagan*, Hiberno. 8vo. Edin. 1791.

20. Dissertatio Inauguralis de Pneumonia. Auctore *Gulielmo Girod*, Anglo. 8vo. Edin. 1791.

21. Dissertatio Inauguralis de Hæmorrhagiis. Auctore *Joanne Langford*, Hiberno. 8vo. Edin. 1791.

22. Dissertatio Inauguralis de Framboesia. Auctore *Jonathan Anderson Ludford*, Jamaicensi. 8vo. Edin. 1791.

23. Dissertatio Inauguralis de Hydrope Anasarca. Auctore *Thoma Story*, Anglo. 8vo. Edin. 1791.

24. Dissertatio Inauguralis de Calculi et Podagræ Nexu. Auctore *Gulielmo Tattersall*, Anglo. 8vo. Edin. 1791.

25. Diſſertatio Inauguralis de Scrofula. Auctore *Jacobo Wood*, Britanno. 8vo. Edin. 1791.

26. Pharmacopœia Collegii Regii Medicorum Edinburgenſis. 8vo. Edin. 1792.

27. De Fœtu humano Diſſertatio Anatomico-Phyſiologica. Auctore *Onuphrio Agneſe Scaſſi*, Genuenſi, Philoſophiæ et Medicinæ Doctore, Regiæ Societatis Medicæ Edin. Membro, Societ. Chirurgo-Phyſicæ Hon. Soc. et Præſide. 8vo. Edinburgi, 1792.

28. Libellus Pharmaceuticus compoſita et præparata præcipua præparandi Modum et Excheireſes exhibens; cui accedunt Tabulæ pro Compoſitionum pharmaceuticarum Proſpectu faciliori: edidit *Johannes Bernardus Keup*, Med. Doct. Urbis et Satrapiæ Solingenſis in Ducatu Montenſi Medicus ordinarius. 8vo. Duiſburg. 1789.

29. *Aloyſii Suareſii Barboſæ*, R. Philoſoph. Profeſſor. emerit. ac Leirenſis Medici, de Angina ulceroſa, ab anno 1786 ad annum 1787 apud Leiriam epidemice graſſante Commentatio. 8vo. Liſbon, 1789.

30. Flora Lipſienſis ſiſtens Plantas in agris Circuli Lipſici tam ſponte naſcentes quam frequentius cultas ſecundum Syſtema ſexuale reviſum atque emendatum deſcriptas a *Joanne Chriſtiano*

*tiano Gottlob Baumgarten*, Med. Bacc. Tabulis IV. aeri inciſis. 8vo. Lipſiæ, 1790.

31. Deſcriptio Febrium acutarum ordinariarum et Febrium catarrhalium ordinariarum, et Dilucidatio Centum et Triginta Aphoriſmorum Hippocratis ad Febres acutas ordinarias pertinentium, data a *Ferd. Saalmann*, Med. Doctore. 4to. Munſter, 1790.

32. Deſcriptio Febrium malignarum in Genere et ſpeciatim ſic dictarum catarrhalium malignarum ſimplicium et exanthematicarum item Petechiarum verarum, deinde Peſtis, ſive Peſtilentiæ veræ, denique Rabiei caninæ, data a *Ferd. Saalmann*, Med. Doct. 4to. Munſter, 1791.

33. *Car. Godofr. Hagen*, Med. D. et Prof. ord. Programma primum de Plantis in Pruſſia cultis. 8vo. Konigſberg, 1791.

34. Collectio Diſſertationum ſelectarum in variis fœderati Belgii Academiis editarum ad omnem Medicinæ Partem pertinentium quam imprimis curavit *W. X. Janſen*. Tom. I. 4to. Duſſeldorf. 1791.

35. *Theodorici Leonhardi Oſkamp*, A. A. L. L. Magiſt. et Phil. Doct. Veteris ad Rhenum Trajecto-Batavi, Diſquiſitio Chemico-Medica de Calcinatione Metallorum per Aquæ Analyſin, eorumeorumque per ejusdem Fluidi Synthesin Reductione. 8vo. Marpurgi. 1791.

36. *Josuæ Baumann*, Wœschbachio—Thurgoviensis Helveti, Miscellanea * Medico-Botanica. 8vo. Marpurgi, 1791.

37. Memoire sur les Moyens de perfectioner l'Etablissement public formé à Lyon en Faveur des Personnes noyées, avec des Remarques sur la Cause de leur Mort, et le Traitement qui leur convient. Par M. *Desgranges*, Medecin et Chirurgien a Lyon, de l'Academie Royale de Chirurgie, et de la Société Royale de Medecine de Paris, des Sociétés Litteraires de Rome, d'Arras, de Valence, de Bourg (au Departement de l'Ain), de Ville Franche, et Chirurgien Major de la Garde Nationale. 4to. Lyon, 1790.

38. Supplement au Memoire sur les Moyens de perfectionner l'Etablissement public formé a Lyon en faveur des personnes noyées, ou l'on demontre de nouveau l'extreme necessité de surveiller cet Etablissement, et ou l'on traite des

* Viz. 1. de utili ac honesto Botanices studio, ex monumentis veterum. 2. De pollinis energia, atque sexu plantarum. 3. De systemate Gleditschii a situ staminum exarato.

moyens de ſtimuler les Organes internes pour les rappeler a leurs Fonctions; ſuivi de Recherches ſur l'Emploi des Lavemens de Fumée de Tabac dans les diverſes Eſpeces d'Aſphyxie, notamment dans celle de Submerſion, et dans le Traitement de pluſieurs autres Maladies; ou Reponſe à la Lettre de *M. Coindre*, du College de Chirurgie de Lyon, Inſpecteur des Secours pour les Noyés. Par *M. Deſgranges*, D. M. et Chirurgien, &c. 4to. Lyon, 1790.

40. Mineralogie Homerique; ou Eſſai ſur les Mineraux dont il eſt fait Mention dans les Poemes d'Homere. Par *Aubin Louis Millin.* 8vo. Paris, 1790.

106. Del vario Modo di curare l'Infezzione Venerea, e ſpecialmente del uſo vario del Mercurio. Storia generale e raggionata di *Pierantonio Perenotti*, di Cigliano, Chirurgo Maggiore del Regimento delle Guardie di ſua Maeſte il Re di Sardegna. 8vo. Turin, 1790.

45. Storia generale e raggionata dell' Origine, dell' Eſſenza o ſpecifica Qualita dell' Infezzione Venerea, di ſua Sede nè Corpi, e de' principall ſuoi Fenomeni; di *Pierantonio Perenotti*, di Cigliano, &c. 8vo. Turin, 1790.

INDEX.

### INDEX.

### A.

ANASARCA, Differtation on, — 222
Andrews, John, Cafe of Injury of the Brain, 12
Angier, Carol. de Rheumatifmo, — 221
Angina. See Sore Throat.
Animal Electricity. See Electricity.
Arbuthnot, Dr. his Obfervations on the Air of Cities quoted, — — — 89
Aretæus, recommends an aftringent Diet in Hæmorrhages, 75
Afthma, fpafmodic, Differtation on, — 222

### B.

Baker, Sir Geo. his Account of a convulfive Difeafe among fome poor Children, — — 99
Barbarofa, A. S. de Angina Ulcerofa, — 223
Barrow, Gulielm. de Variolis, — 221
Baumann, Jof. Mifcellanea Medico-Botanica, 225
Baumgarten, J. C. Flora Lipfienfis, — 223
Bengal, a Work on the Fevers and Dyfentery of, 220
Bradley, Tho. de Epifpafticis, — 221
Brain, Injury of, without a Fracture, relieved by the Trephine, — — — 12
Briggs, Gul. de Ictero, — — 221
Butter, Dr. W. on the Angina Pectoris, — 220

### C.

Calculus, urinary, Experiments on, — 121
————, Differtation on its Connexion with the Gout, — — 222
Caneftrini, Ant. Cafe of double Uterus, — 171
Catalepfy, hyfterical, Inftance of, — 52
Cataract, Treatife on, — — 219
Chorin, M. Cafe of double Hare Lip, — 153
Clarke, Dr. Jofeph, on a Difeafe which has been very fatal to Infants in the Dublin Lying-in Hofpital, 78
Cleghorn, Dr. his Account of the Trifmus nafcentium, 96
Clendining, Joann. de Afthmate fpafmodico, 222

Copper, Remarks on the Poifon of, —— 66

Cow, Cafe of Hydatids extracted from the Brain of one, 17

Cullen, Dr. his Practice in Hæmoptyfis objected to, 76

D.

Davidfon, W. Cafe of a fingular cutaneous Affection, 61
————, Remarks relative to the Poifon of Copper, 66
————, Cafes of pulmonary Hæmorrhage, 68
Defgranges, M. fur les Noyés, —— 225
Douglafs, Adam, de Epilepfia, —— 222
Dreams, Differtation on, — — *ibid.*
Drowned Perfons, Works relative to the Recovery of, 224
———— Animals, fometimes recovered by exciting their Electricity, —— —— 217
Dublin Lying-in Hofpital, Account of a Difeafe that has been fatal to Infants there, —— 78
Dyfpepfia, Differtation on, — — 222

E.

Electricity, its Efficacy in a Cafe of fpafmodic Affection, 52
————, animal, how firft difcovered, — 180
————, Exp. relative to, by Prof. Galvani, 180
————, by Dr. Valli, 191, 212
Elliot, Phil. de Dyfpepfia, —— 222
Encyfted Tumours, the Nature of, confidered, 114
Epilepfy, Differtation on, — — 222
Epifpaftics, Differtation on, —— 221
Excifion recommended as the only certain Preventative of Hydrophobia, —— —— 33
Excrefcences, horny, of the human Body, Obfervations on, 103
————, Inftances of, 104, 107, 108, 109, 110, 111
————, their Mode of Formation explained, 112

F.

Fits, nine-day, a Difeafe of Infants fo called, 80
————, Fatality of, in the Lying-in Hofpital at Dublin, — — — *ibid.*
————, Hiftory of, — — 81
————, Obfervations on the Caufes of, 83, 102
————, Succefs of Ventilation in preventing them, 90

Fits,

Fits, nine-day, Remarks on their nosological Arrangement, 95

————, their Affinity to the Trism. Nascentium, 96

Fœtus, human, Dissertation on, — 223

Foot, Jesse, on the Prevention of Hydrophobia, 33

Fordyce, Sir W. on the Cultivation of Rhubarb, 221

Frambroesia. See Yaws.

G.

Gahagan, Jos. de Somniis, — 222
Galvani, Lewis, Experiments on animal Electricity, 180
Garnett, Dr. Thomas, on the Horley-green Spa, 219
Girod, Gulielm. de Pneumonia, — 222
Gout, Dissertation on its Connexion with urinary Calculus, 222

H.

Hæmorrhages, Dissertation on, — 222

————, pulmonary, Remarks on, — 68

————, to be restrained by Abstinence from Liquids, — — 75

Hagen, C. G. de Plantis Prussicis, — 224

Hare Lip, Case of, — — 153

————, Observations on the Cure of, — 161

Heister, his Observ. on Spasm of the Jaw in Children, 96

Hofer, his Account of a Species of Tetanus in Children, 97

Home, Ev. on horny Excrescences of the human Body, 103

Homer, Essay on the Minerals mentioned by, - 226

Horny Excrescences. See Excrescences.

Houlston, W. his Edition of Mr. Justamond's Works, 218

Hughes, T. Cases of Fracture of the Jaw, — 36

Hunter, Mr. his Theory of the Nature of encysted Tumours, — — 115

Hydatids extracted from the Brain of a Cow, Case of, 17

————, different Species of, defined, — 19

Hydrophobia, Facts relative to the Prevention of, 33

I.

James's Powder, its Composition investigated, 128

———— its sensible Properties, — *ibid.*

———— specific Gravity, — 129

———— the Ingredients of, enumerated, 151

———— for Horses, a Preparation sold under that Name, described, — — 152

Janfen, W. X. Collectio Differtationum, — 224
Jaundice, Differtation on, ——— 221
Jaw, Cafes of Fracture of, ——— 36
Jebb, Dr. John, his Defcription of a Cafe of Catalepfy quoted, ——— ——— 55
Infants, Mortality of, in different Places, ——— 85
Ifchuria Renalis in Children, Cafes of, ——— 1
———————, Symptoms of, - *ibid.*
———————, afcribed to mefenteric Inflammation, ——— ——— 7
Juftamond, J. O. Surgical Tracts, ——— 218

K.

Keup, J. B. Libellus Pharmaceuticus, ——— 223

L.

Lane, Timothy, Experiments on Human Calculi 121
Langford, Joann. de Hæmorrhagiis, ——— 222
Latham, Dr. John, Plan of a Charitable Inftitution for Sea Bathing, ——— ——— 221
Lieutaud, M. his Opinion of Catalepfy, — 61
Lile's Fever Powder, Account of, ——— 138
Linnean Society, Tranfactions of, ——— 220
Liquids, Abftinence from, recommended in pulmonary Hæmorrhage ——— ——— 75
Ludford, J. A. de Framboefia, ——— 222
Lungs, Remarks on their Structure, ——— 77
Lying-In Hofpitals, confidered as Inftitutions of recent Date, 78

M.

Mefenteritis, Defcription of, by Riverius, ——— 8
Millin, A. L. Mineralogie Homerique, — 226
Minorca, Account of the *Trifmus Nafcentium* as it prevails there, ——— ——— 96
Moorcroft, William, Cafe of Hydatids, extracted from the Brain of a Cow, ——— ——— 17
Morlen, William, Cafe of an enlarged Nympha, 50
Mufcular Motion, fhewn to depend on Electricity, 180

N.

Nympha, enlarged, Cafe of, ——— 50

O.

Oeftrus, different Species of mentioned, —— 23
Ofkamp, Th. Leon. de Calcinatione Metallorum, 224

P.

Pearfon, Dr. Geo. Experiments on James's Powder, 128
Pemphigus, Cafe of, —— —— 10
Perenotti, P. dell' Infezzione Venerea, —— 226
Pharmacopœia, Colleg. Reg. Med. Lond. — 221
———————————— Edin. — 225
———— Mancunienfis, — — 221
Pneumonia, Differtation on, — — 222
Pole, Thomas, the Anatomical Inftructor, — 219
Polydipfia, Cafe of, —— —— 167

R.

Rheumatifm, Differtation on, — — 221
Rhinoceros, Remarks relative to its Horn, — 117
Riverius, his Defcription of Mefenteritis, — 8
Rowley, Dr. recommends Abftinence from Liquids in Menorrhagia, —— —— 76

S.

Saalmann, Ferd. Defcriptiones febrium acutarum et malignarum, —— —— 224
Scaffi, O. A. de Fœtu Humano, — — 223
Schawanberg's Fever Powder, Account of, — 138
Schrœder's Fever Powder, Account of, —— *ibid.*
Scrofula, Differtation on, —— — 223
Severinus, recommends the Biftoury in Cafes of Hare Lip, 162
Short, Dr. his Obfervations on the Mortality of Children in different Places, — — 85
Small Pox, Differtation on, —— — 221
Sore Throat, ulcerated, Work relative to, — 223
Sponge, its Utility in certain Circumftances of fractured Jaw, — — — — 32. 44
Stone. See Calculus.
Story, Tho. de Anafarca, — — 222
Suture in the Operation for the Hare Lip, Obfervations on, 163

### T.

Tatterfall, Gul. de Calculi et Podagræ Nexu, 222
Tetanus, Inftance of as an Hyfterical Affection, 56
——— in Infants. See Trifmus.
Thirft, Exceffive, Cafe of, — — 167
——————— Theory of the Caufes of, — 169
Trifmus Nafcentium, Obfervations relative to, 96, 97, 98

### U.

Valli, Dr. Eufebius, Obfervations on Animal Electricity, 191, 212
Vaffalli, Abbé, his Conjectures relative to Animal Electricity, — — — 180
Vauquelin, M. Cafe of a Child who drinks a great Quantity of Water, —— —— 167
Ventilation, good Effects of, in the Lying-In Hofpital at Dublin, ——— —— 90
Venereal Difeafe, Works relative to it. — 226
Urine, Analyfis of in a Cafe of Polydipfia, — 169
Uterus, Double, Cafe of, — — 171

### W.

Wade, Dr. J. P. on the Fever and Dyfentery of Bengal 220
Ware, James, a Treatife on the Cataract, — *ibid.*
Waters, Mineral, Work relative to, —— 219
Weeks, J. C. Facts relative to Hydrophobia, — 35
Wenzel, Baron de, on the Cataract, — 220
Wilkinfon, Geo. Cafe of Spafmodic Affection, - 52
Willan, Dr. Robert, Cafes of Ifchuria Renalis, - 1
Winterbottom, Dr. T. M. Cafe of Pemphigus, 10
Wood, Jac. de Scrofula, — — 223

### Y.

Yaws, Differtation on, — — 222

END OF THE THIRD VOLUME.

